# Disappearance of Oral Lichen Planus After Liver Transplantation for Primary Biliary Cirrhosis and Immunosuppressive Therapy in a 63-year-Old Japanese Woman

**DOI:** 10.5812/hepatmon.16310

**Published:** 2014-03-11

**Authors:** Yumiko Nagao, Michio Sata

**Affiliations:** 1 Department of Digestive Disease Information and Research, Kurume University School of Medicine, Kurume, Japan; 2 Division of Gastroenterology, Department of Medicine, Kurume University School of Medicine, Kurume, Japan

**Keywords:** Lichen Planus, Oral, Liver Cirrhosis, Biliary, Liver Transplantation

## Abstract

**Introduction:**

There are few reports concerning association between primary biliary cirrhosis (PBC) and lichen planus. In addition, there is only one report about lichen planus after liver transplantation.

**Case Presentation:**

We describe a case of oral lichen planus (OLP) accompanied with PBC that resolved following liver transplantation 14 years later. This patient received immunosuppressive drugs after liver transplantation.

**Discussion:**

The disappearance of OLP might be due to immunosuppressive therapy following liver transplantation. Further observations and studies are necessary to clarify the relationship between OLP and PBC.

## 1. Introduction

The association between oral lichen planus (OLP) and hepatitis C virus (HCV) infection has been reported frequently (1, 2). In Japan, the rate of HCV infection in OLP is especially high (3). On the other hand, the association between lichen planus and primary biliary cirrhosis (PBC) been reported rarely since Seehafer et al. pointed it out in 1981 (4). PBC is a chronic cholestatic liver disease of adults. Progressive bile-duct injury from portal and periportal inflammation leads to progressive fibrosis and eventually, cirrhosis (5). Evidences to date suggest that the disease might be caused by immunological and genetic factors. Affected individuals are typically middle-aged women with asymptomatic rises of serum biochemical markers of hepatic injury.

An epidemiological study from United States showed that one-third of 1032 patients with PBC were affected by another autoimmune disease, most commonly Sjögren's syndrome, Raynaud’s phenomenon, autoimmune thyroid disease, scleroderma, or systemic lupus erythematosus (SLE) (6). Oleaga et al. previously reported a case of generalized lichen planus associated with PBC, which cleared after liver transplantation (7). We report a case of OLP accompanied by PBC that disappeared after liver transplantation.

## 2. Case Presentation

**Figure 1.**
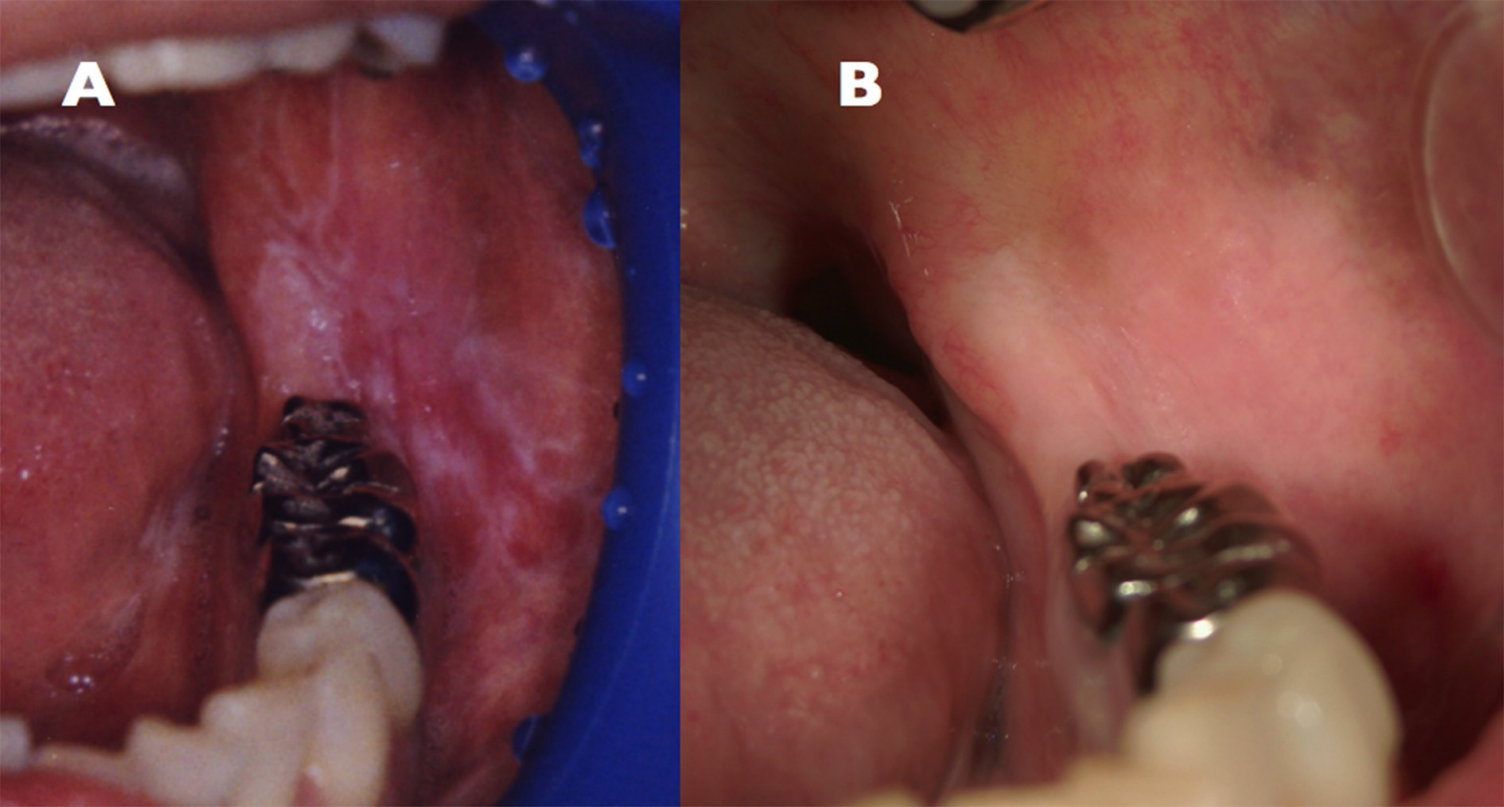
(A) OLP Lesions Affecting the Bilateral Buccal Mucosa (January 1995), (B) Disappearance of OLP (May 2011)

In January 1995, a 45 year old Japanese female presented at the Kurume University Hospital (Fukuoka, Japan) with a burning pain in the bilateral buccal mucosa on eating and drinking. The lesion was diagnosed as OLP by biopsy (Figure 1 A). The biopsy specimen was characterized by hyperparakeratosis with thickening of the granular layer, a subepithelial band of infiltration of lymphocytes and liquefied degeneration of the basal cell layer. The histopathological findings were consistent with a diagnosis of OLP. The erosive OLP lesion was not widely aggravated by application of steroids. She consulted an oral surgeon at our hospital every six months to detect chenges in the oral lesion.

Concerning her history, she had been hospitalized for aggravation of liver function tests at the ages of 20 (in 1970) and 23 (in 1973). In August, 1992, the 42-year-old patient was admitted to a nearby hospital, again because of aggravation of liver function and icterus. Data from that time were obtained from her family doctor included aspartate aminotransferase (AST) 547 U/L (normal range, 13-33), alanine aminotransferase (ALT) 818 U/L (normal range, 6-30), total bilirubin (T. Bil) 10.5 mg/dL (normal range, 0.30-1.20), albumin (Alb) 3.3 g/dL (normal range, 4.00-5.00), negative results for anti-HCV antibody (anti-HCV), serum HCV RNA, hepatitis B surface antigen (HBsAg), IgM antibody to hepatitis B core antigen (IgM anti-HBc), hepatitis B surface antibody (anti-HBs), IgM antibodies to hepatitis A virus (IgM anti-HAV), antimitochondrial antibodies (AMA), anti-smooth muscle antibody (SMA), liver kidney microsome antibody type 1 (LKM-1), and positive results for anti-nuclear antibody (ANA). After hospitalization, she was treated with oral ursodeoxycholic acid (UDCA) by her family doctor for liver cirrhosis caused by autoimmune hepatitis (Table 1). The patient did not receive systemic steroid therapy.

Hypertension was noted at the age of 42 years and treatment with an antihypertensive drug was started. In addition, she was diagnosed with Sjögren's syndrome at the age of 42 years. There was no history of blood transfusion or tattooing and her family history was not contributory. The laboratory data at her first visit for examination of oral membrane disease in 1995 were AST 31 U/L, ALT 23 U/L, gamma-glutamyl transpeptidase (gamma-GTP) 34 U/L, total protein (T. pro) 7.4 g/dL, Alb 4.1 g/dL, T. Bil 1.5 mg/dL, direct bilirubin (D. Bil) 0.8 mg/dL, negative results for anti-HCV, serum HCV RNA, and HBsAg (Table 1). 

Because the patient had a progressive worsening of her symptoms including fatigue and pruritus around June 2008 (at 58-years-old), she was admitted to our hospital for a detailed evaluation of her liver disease in June 2009. The investigations findings included elevation of ALP and IgM but negative results for AMA, complication of osteoporosis with biliary stasis, compression fractures of the third and fourth lumbar vertebrae from osteoporosis, complication of Sjögren's syndrome and chronic thyroiditis, pruritus, and erosive OLP. Based on these findings, she was diagnosed as progressive PBC for the first time. The likelihood of the patient's 6-month survival was 22%, in accordance with The Updated Natural History Model for PBC (8, 9). The updated Mayo model includes five independent variables predicting survival: age, serum levels of bilirubin and albumin, prothrombin time, and the presence or absence of peripheral edema, including response to diuretic therapy.

This patient was treated as an inpatient by branched chain amino acids (BCAA), UDCA, diuretic drug, and antithyroid agent. The symptom of OLP did not aggravate. She had some teeth extracted for chronic apical periodontitis and received liver transplantation from a living donor in September 2009. Treatment with immunosuppressive agents such as tacrolimus (Prograf®) and mycophenolate mofetil (CellCept®) was started. In May 2011, the symptoms of OLP disappeared (Figure 1 B). In addition, the clinical condition of PBC improved (Table 1). 

**Table 1.** Summary of the Clinical Condition of Oral Lesion and Laboratory Data

	Normal Range	On Admission to a Previous Hospital	First Visit to Examine Oral Lesions	On Admission to This Hospital	Liver Transplantation	After Liver Transplantation	Recent Visit
**Date**		Aug 21, 1992	Jan 17, 1995	Jun 23, 2009	Sep 1, 2009	May 17, 2011	Aug 27, 2013
**Age, y**		42 years old	45 years old	59 years old	59 years old	61 years old	63 years old
**Clinical Condition of Oral Lesion**		unknown	OLP ^a^ presence, biopsy	OLP presence		OLP disappearance	OLP disappearance
**Laboratory Data**							
**AST ** ^**a**^ ** ,U/L**	13-33	547	31	53		21	25
**ALT ** ^**a**^ ** , U/L**	6-30	818	23	25		13	19
**T. pro ** ^**a**^ ** , g/dL**	6.70-8.30	10.5	7.4	5.84		7.45	7.08
**Alb ** ^**a**^ ** , g/dL**	4.00-5.00	3.3	4.1	2.34		4.86	4.41
**gamma GTP ** ^**a**^ ** , U/I**	10-47	unknown	34	33		22	16
**ALP ** ^**a**^	115-359	unknown	ND	513		238	141
**T. BIl ** ^**a**^ ** , mg/dL**	0.30-1.20	10.50	1.50	10.70		1.33	0.94
**D.Bil ^a^, mg/dL**	≤ 0.60	unknown	0.80	6.71		0.18	0.13
**T. C ** ^**a**^ ** ,mg/dL**	128-219	95	ND ^a^	97		227	209
**Hb ^a^, g/dL**	11.0-15.0	unknown	ND	11.9		14.5	13.7
**PLT a, × 10 ^4^/μL**	13.0-36.0	unknown	ND	7.0		11.4	15.5
**AFP ^a^, ng/mL**	≤ 8.7	unknown	ND	3.2		4.5	4.0
**IgA ^a^, g/dL**	103-409	unknown	ND	274		ND	ND
**IgM ^a^, mg/d**	40-221	unknown	ND	236		ND	110
**IgG ^a^, mg/dL,**	918-1742	unknown	ND	1309		ND	ND
**FT4 ^a^, ng/dL**	0.88-1.56	unknown	ND	1.27		ND	ND
**TSH ** ^**a**^ ** , µIU/L**	0.210-3.850	unknown	ND	5.040		ND	ND
**Anti-HCV ** ^**a**^		negative	negative	negative		ND	ND
**Serum HCV RNA**		negative	negative	negative		ND	ND
**HBsAg ** ^**a**^		negative	negative	negative		ND	ND
**IgM anti-HBc ** ^**a**^		negative	ND	ND		ND	ND
**Anti-HBs ** ^**a**^		negative	ND	ND		ND	ND
**Anti-HBc ** ^**a**^		ND	ND	negative		ND	ND
**IgM anti-HAV ** ^**a**^		negative	ND	ND		ND	ND
**AMA ** ^**a**^		negative	ND	ND		ND	ND
**SMA ** ^**a**^		negative	ND	negative		ND	ND
**LKM-1 ** ^**a**^		negative	ND	negative		ND	ND
**ANA ** ^**a**^		positive	ND	positive		ND	ND

^a^ Abriviations: AFP, alpha-fetoprotein; Alb, albumin; ALP, alkaline phosphatase; ALT, alanine aminotransferase; AMA, antimitochondrial antibodies; ANA, anti-nuclear antibody; Anti-HBs, hepatitis B surface antibody; Anti-HBc, hepatitis B core antibody; Anti-HCV, anti-HCV antibody; AST, aspartate aminotransferase; D.Bil, direct bilirubin; FT4, free thyroxine 4; gamma GTP, gamma-glutamyl transpeptidase; Hb, hemoglobin; HBsAg, hepatitis B surface antigen; IgM anti-HBc, IgM antibody to hepatitis B core antigen; IgM anti-HAV, IgM antibodies to hepatitis A virus; LKM-1, liver kidney microsome antibody type 1; ND, not detected; PLT, platelets; T.pro, total protein; T.BIl, total bilirubin; T.C, total cholesterol; TSH, thyroid stimulating hormone; SMA, anti-smooth muscle antibody

## 3. Discussion

PBC patients present with a wide variety of cutaneous manifestations, varying in severity (10). Koulentaki et al. reported that the incidence rates of cutaneous lichen planus and OLP in patients with PBC were 10.2% (5/49 cases) and 8.2% (4/49), respectively (10). Powell et al. reported that the incidence rates of lichen planus in PBC without D-penicillamine (d-PCN) therapy and after d-PCN therapy were 2.6% (7/268 cases) and 12.9% (17/131), respectively (11). In Japan, there are no \epidemiologic studies concerning association between PBC and lichen planus.

Liver transplantation can be successful in treating end-stage liver disease from PBC (12). The survival of liver transplant recipients with PBC diminishes significantly at risk scores above 7.8 (13). Following transplantation, the majority of patients receive immunosuppressive therapy consisting of combinations of corticosteroids, azathioprine, cyclosporine, and tacrolimus.

In previous studies, the effect of immunosuppressive agents such as tacrolimus and mycophenolate mofetil on severity and progression of OLP were repoted (14-16). Tacrolimus inhibits the activation and proliferation of T-lymphocytes by inhibiting the phosphatase activity of calcineurin and mycophenolate mofetil inhibits lymphocyte proliferation and activation. However, Lodi et al. reported that these agents theoretically could also trigger malignant transformation (17). It has been reported that the topical use of tacrolimus in patients with OLP may promote the development of squamous cell carcinoma (18). For example, cyclosporin can promote cancer progression, both by a direct cellular effect and by an effect on the host’s immune system (19).

In a retrospective study, Wimmer et al. analyzed the development of de novo malignancy after successful liver transplantation of 609 patients (20). The most frequent observed tumors were non-melanoma skin cancers (44.83%). Moreover, post-transplant lymphoid disease, oropharyngeal cancer (n = 6, 6.9%), upper gastrointestinal tract cancer (n = 4, 4.6%), lung cancer (n = 4, 4.6%), gynecological malignancies (n = 4, 4.6%), and kidney cancer (n = 3, 3.45%) were detected. Multivariate analysis revealed recipient age (hazards ratio (HR) 1.06), male gender (HR 1.73), and tacrolimus-based immunosuppression (HR 2.06) as significant risk factors.

In conclusion, immunosuppressive therapy following liver transplantation for PBC led to the disappearance of OLP lesion in our patient as was previously reported by Oleaga et al. (7). Patients with PBC are at increased risk of occurrence of hepatocellular carcinoma and extrahepatic malignancies (21). Long-term follow-up is needed to elucidate the therapeutic effects of transplantation.
